# Supplementary material for: The IAA-Producing Rhizobacterium Bacillus sp. SYM-4 Promotes Maize Growth and Yield
Source: Plants (Basel). 2025 May 23;14(11):1587. doi: 10.3390/plants14111587 (PMC12158203; doi:10.3390/plants14111587)
Supplement: Supplementary file 1 [file plants-14-01587-s001.zip › plants-3625633-supplementary.pdf]

The IAA producing rhizobacterium *Bacillus* sp. SYM-4 with promotes maize growth and yield

Supplementary materials

Yumeng Song#, Qifei Chen#, Juan Hua, Shaobin Zhang*, Shihong Luo*

Engineering Research Center of Protection and Utilization of Plant Resources, College of Bioscience and Biotechnology, Shenyang Agricultural University, Shenyang, 110866, Liaoning Province, China

#These authors have made equal contributions.

*Corresponding Authors

Dr. Shaobin Zhang, zsb@syau.edu.cn; Prof. Shihong Luo, E-mail address: luoshihong@syau.edu.cn (http://orcid.org/0000-0003-3500-3466)

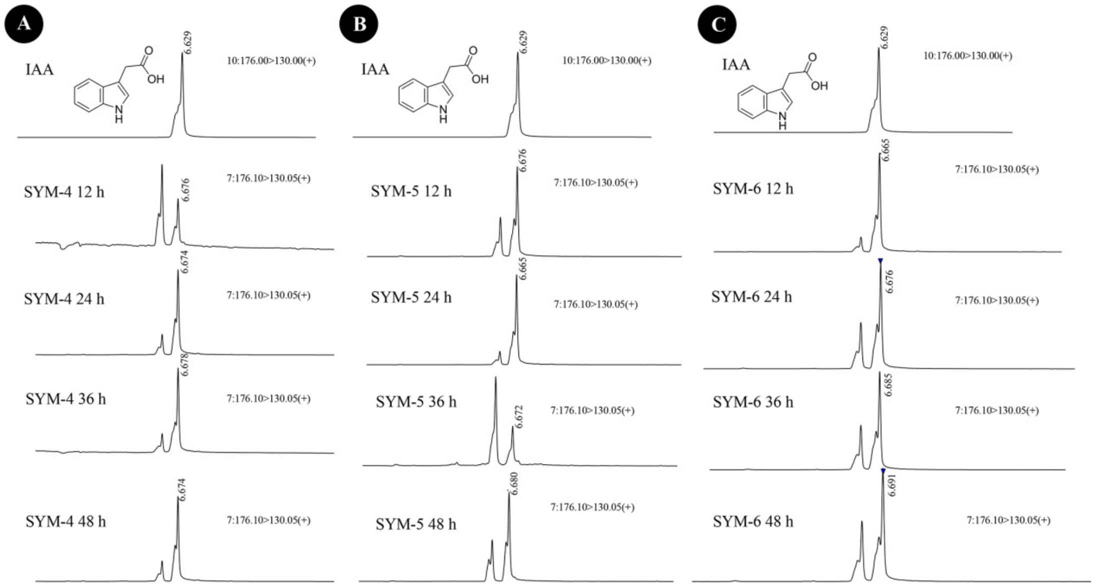

Figure S1. Qualitative and quantitative UPLC-MS/MS analysis of IAA synthesis in SYM-4 (A), SYM-5 (B), and SYM-6 (C) strains activated for 12-48 h.

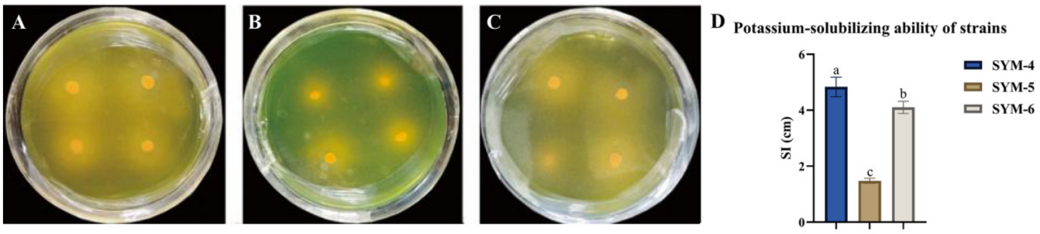

Figure S2. Determination of potassium-releasing activity of SYM-4 (A), SYM-5 (B) and SYM-6 (C). Different letters above the graph bars in part D indicate significant differences between values.

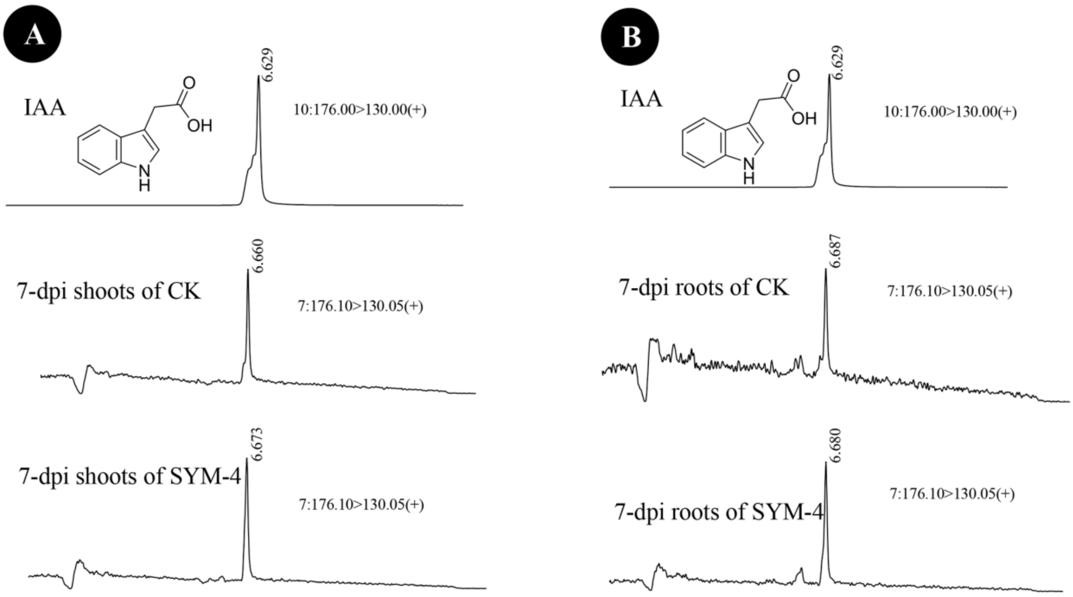

Figure S3. The qualitative and quantitative UPLC-MS/MS determination of IAA in the shoots (A) and roots (B) of maize seedlings grown for 7 days following inoculation with *Bacillus* sp. SYM-4.

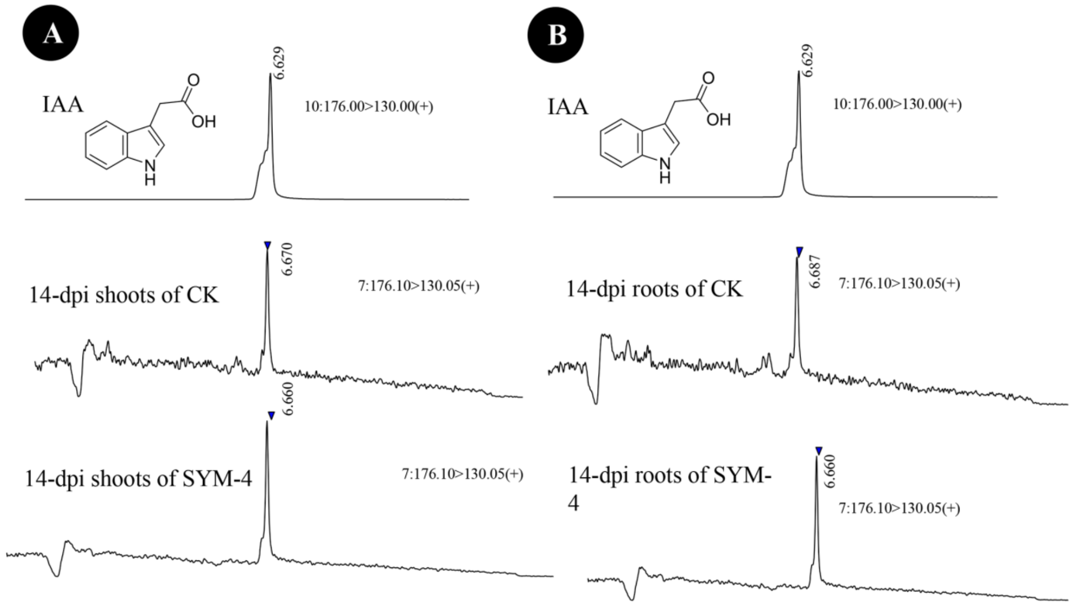

Figure S4. The qualitative and quantitative UPLC-MS/MS determination of IAA in the shoots (A) and roots (B) of maize seedlings grown for 14 days following inoculation with *Bacillus* sp. SYM-4.

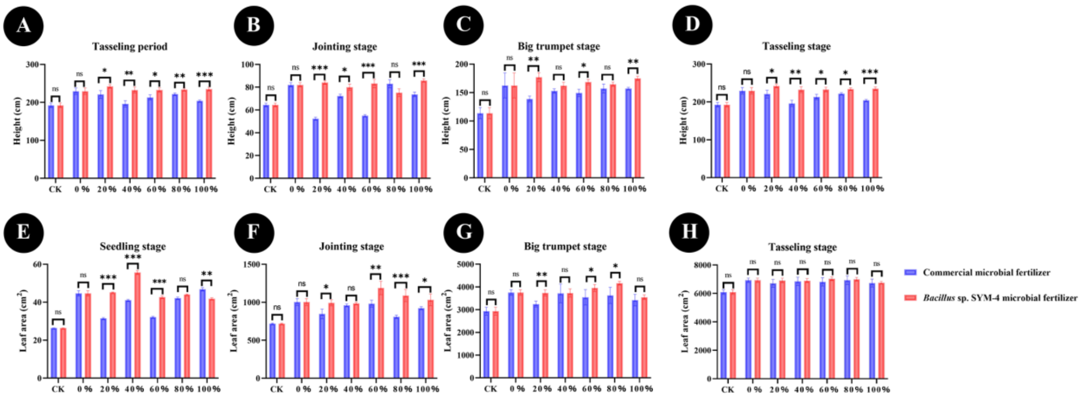

Figure S5. Plant height and leaf area of maize at four growth stages grown following application of fertilizer in which different proportions of a chemical fertilizer were replaced with either *Bacillus* sp. SYM-4 microbial fertilizer or a commercial microbial fertilizer.

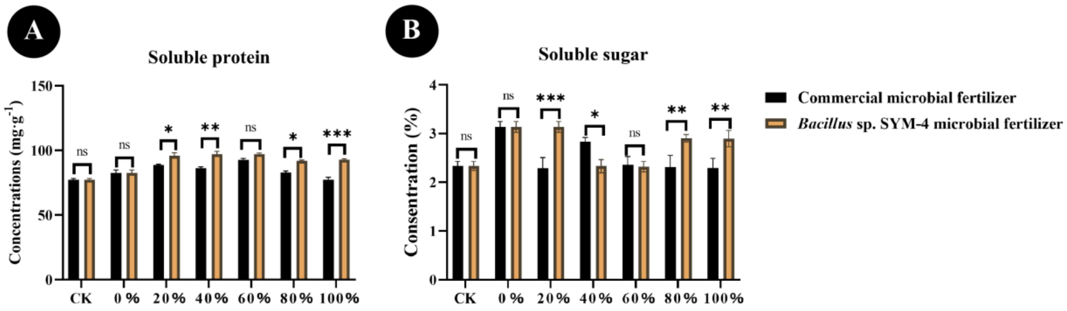

Figure S6. The soluble protein (A) and soluble sugar (B) content in the maize kernels in maize seedlings grown in the field following application of a fertilizer in which different proportions of a chemical fertilizer replaced with either *Bacillus* SYM-4 microbial fertilizer or a commercial microbial fertilizer.
